# Does stapes surgery improve tinnitus in patients with otosclerosis?^☆^

**DOI:** 10.1016/j.bjorl.2016.07.001

**Published:** 2016-08-02

**Authors:** Onur Ismi, Osman Erdogan, Mesut Yesilova, Cengiz Ozcan, Didem Ovla, Kemal Gorur

**Affiliations:** a University of Mersin, Faculty of Medicine, Department of Otorhinolaryngology, Mersin, Turkey; b University of Mersin, Faculty of Medicine, Department of Biostatistics, Mersin, Turkey

**Keywords:** Otosclerosis, Tinnitus, Stapedotomy, Low pitch, High pitch, Otosclerose, Zumbido, Estapedotomia, Grave, Agudo

## Abstract

**Introduction:**

Otosclerosis (OS) is the primary disease of the human temporal bone characterized by conductive hearing loss and tinnitus. The exact pathogenesis of tinnitus in otosclerosis patients is not known and factors affecting the tinnitus outcome in otosclerosis patients are still controversial.

**Objectives:**

To find the effect of stapedotomy on tinnitus for otosclerosis patients.

**Methods:**

Fifty-six otosclerosis patients with preoperative tinnitus were enrolled to the study. Pure tone average Air-Bone Gap values, preoperative tinnitus pitch, Air-Bone Gap closure at tinnitus frequencies were evaluated for their effect on the postoperative outcome.

**Results:**

Low pitch tinnitus had more favorable outcome compared to high pitch tinnitus (*p* = 0.002). Postoperative average pure tone thresholds Air-Bone Gap values were not related to the postoperative tinnitus (*p* = 0.213). There was no statistically significant difference between postoperative Air-Bone Gap closure at tinnitus frequency and improvement of high pitch tinnitus (*p* = 0.427). There was a statistically significant difference between Air-Bone Gap improvement in tinnitus frequency and low pitch tinnitus recovery (*p* = 0.026).

**Conclusion:**

Low pitch tinnitus is more likely to be resolved after stapedotomy for patients with otosclerosis. High pitch tinnitus may not resolve even after closure of the Air-Bone Gap at tinnitus frequencies.

## Resumo

**Introdução:**

Otosclerose (OS) é a principal doença do osso temporal humano caracterizada por perda auditiva condutiva e zumbido. A patogenia exata do zumbido em pacientes com otosclerose não é conhecida e fatores que afetam o desfecho de zumbido em pacientes com otosclerose ainda são controversos.

**Objetivos:**

Encontrar o efeito da estapedotomia sobre o zumbido em pacientes com otosclerose.

**Método:**

Foram incluídos no estudo 56 pacientes com otosclerose com zumbido pré-operatório. Os valores médios tonais do gap aero-ósseo, o tom de zumbido no pré-operatório, o fechamento do gap nas frequências dos zumbidos foram avaliados quanto ao seu efeito sobre o desfecho pós-operatório.

**Resultados:**

O zumbido em tom grave teve desfecho mais favorável em comparação com o zumbido agudo (*p* = 0,002). Os valores médios dos gaps pós-operatórios não foram relacionados com o zumbido pós-operatório (*p* = 0,213). Não houve diferença estatisticamente significativa entre o fechamento pós-operatório do gap na frequência do zumbido e melhora do zumbido de tom agudo (*p* = 0,427). Houve diferença estatisticamente significativa entre a melhora no gap nas frequências do zumbido e recuperação do zumbido de tom mais grave (*p* = 0,026).

**Conclusão:**

O zumbido de tom mais grave parece ser melhor resolvido depois de estapedotomia em pacientes com otosclerose. O zumbido de tom agudo pode não desaparecer, mesmo após o fechamento do gap nas frequências do zumbido.

## Introduction

Otosclerosis (OS) is the primary disease of the human temporal bone. It is an autosomal-dominant hereditary disease with variable penetrance. Clinically, progressive conductive hearing loss and tinnitus are the main symptoms. The most common area for stapedial fixation is the anterior crura. Sensorineural hearing loss can develop if the plaques involve the cochlea.^1^

Otosclerosis was described about two centuries ago; however, the exact pathogenesis is not fully understood. Although hearing AIDS and medical therapy have been recommended in certain conditions, small fenestra stapedotomy still remains the main choice in patients with conductive type hearing loss.^1^ The success of stapes surgery in patients with OS is evaluated with the results of postoperative hearing and the rate of Air-Bone Gap closure.

Tinnitus is also a common and underestimated symptom beside conductive hearing loss in OS.^2^ Satisfaction rates after stapes surgery is directly related to the postoperative tinnitus cessation. OS patients with unresolved tinnitus in the postoperative period reported significantly lower satisfaction scores from surgery.^3^ The estimated incidence of subjective tinnitus in OS patients is 56–84.5%.^2^, ^4^

The exact pathogenesis of tinnitus in OS is not known. Several authors have reported different possible mechanisms as follows: reduction of inner ear fluid vibration, unmasked muscular or vascular noises with conductive hearing loss, intravascular agglutination of red blood cells in the vessels of the cochlea, toxic metabolites produced by the otosclerotic foci, pathological vascularization of the otosclerotic bone and irritation of the nerve fibers by otosclerotic bone.^4^, ^5^

Although there have been several published studies, the effect of stapes surgery on tinnitus is still a subject of debate. Some authors reported that preoperative low pitch tinnitus is more likely to be resolved by stapes surgery,^5^, ^6^ whereas Gersdorff et al.^7^ and Ayache et al.^8^ found no statistical significance between low and high pitch tinnitus recovery. Apart from tinnitus frequency, preoperative non-compensated tinnitus^9^ and postoperative high frequency hearing loss^3^ were claimed to have an unfavorable outcome. Furthermore, the effect of Air-Bone Gap (ABG) closure at tinnitus frequencies on the postoperative tinnitus outcome has not been discussed in any of the studies.

Different from other studies, in this manuscript, we evaluated the effect of Air-Bone Gap closure at tinnitus frequencies on the low, middle and high pitch tinnitus status.

## Methods

Local ethical committee approval was acquired for our study with the number of 2016190. Sixty-nine patients with conductive hearing loss were enrolled to present study. After a detailed otorhinolaryngologic examination, hearing loss type and hearing thresholds were evaluated regarding tuning fork tests and audiometric findings. The patients with hearing loss (ABG ≥ 20 dB and Rinne [–] with 512, 1024 tuning forks) with speech discrimination scores of ≥90% were operated. Preoperative temporal bone computerized tomography was performed to all patients. The diagnosis of OS was confirmed during surgery. All of the patients underwent small fenestra stapedotomy and hearing restoration with Teflon piston by single surgeon. Patients having other possible etiology for tinnitus such as acoustic trauma, blood biochemistry abnormalities including anemia, vitamin B12 deficiency, thyroid function test abnormalities, and previous ear surgery history were excluded from the study.

### Surgical technique

All patients were operated under the general anesthesia with endaural incision. Briefly; after modified Rosen incision, tympanomeatal flap elevation and chordal bony removal with a curette or drill, mobility of ossicular chain was inspected and palpated to establish the diagnosis. All patients were operated using Fisch's reversal steps stapedotomy technique.^10^ The distance between lateral surface of the incus long process and stapes footplate was measured with a malleable measuring rod. Small fenestra was performed with 0.5 or 0.7 mm drills. Meticulous care was taken to prevent the aspiration of the fenestra and placement of the prosthesis immediately after forming the fenestra. Small fenestra was not sealed before placement of the prosthesis. The mobility of prosthesis is assessed by gentle palpation of malleus.

### Patient characteristics and planning

There were 42 (60.8%) females and 27 (39.1%) males. Mean age was 42 years (range 32–57 years). 56 (81.1%) patients had preoperative tinnitus complaint. The preoperative tinnitus frequency was measured by the pitch-matching test, was calibrated as follow: High frequencies (4 kHz, 6 kHz, and 8 kHz), middle frequencies (1 kHz, 2 kHz, and 3 kHz) and low frequencies (125 Hz, 250 Hz, and 500 Hz).^11^ For pitch-match testing the adaptive (bracketing) method was used. Briefly, the tone was set successively to 9 audiometric frequencies between 0.125 and 8 kHz (0.125, 0.25, 0.5, 1, 2, 3, 4, 6, and 8 kHz), and the subject was asked to indicate which of these frequencies most closely matched the pitch of their tinnitus. The frequency of the test tone was then adjusted in half-octave steps above and below the selected frequency, and the subject was asked to indicate which of the frequencies best matched the tinnitus pitch. The final match was taken as the frequency that the subject judged to match most closely their tinnitus. This technique is recommended for routine clinical use and seems to produce fewer octave errors than other procedures.^12^, ^13^

These patients were divided in four groups regarding their postoperative tinnitus status. Group I, completely recovered; Group II, Improved; Group III, Unchanged; Group IV, Worsened. Group I and II were classified in the same group as “favorable” outcome. Preoperative and postoperative first year pure tone air and bone conduction thresholds, Air-Bone Gap rates were assessed according to the American Academy of Otolaryngology Head and Neck Surgery Guidelines.^14^ Air-Bone Gap was calculated with subtraction of the bone conduction threshold levels from air conduction threshold levels. Success of the surgery was defined as postoperative first year pure tone ABG < 10 dB without sensorineural hearing loss (postoperative > 10 dB deterioration of bone conduction levels compared to preoperative levels for frequencies 1.2 and 4 kHz).^8^

### Statistical analysis

Descriptive statistics were presented as proportions or medians (25–75% percentiles) as appropriate.

All continuous measurements were tested for normality using the Kolmogorov–Smirnov and Shapiro–Wilk test. *p* < 0.05 was accepted as significant. As measurements did not display a normal distribution, non-parametric method Mann–Whitney *U*-test was used for independent groups’ comparison. The comparison results were displayed with the box-plot graph.

Categorical data were compared using pearson Chi-Square with Yates's correction, likelihood ratio or Fisher's exact test. Two proportion *Z* test was used to compare the two groups whether any differences between them were significant.

## Results

Fifty-six (81.1%) of 69 patients had preoperative tinnitus complaint. The overall success rate was 79.7% (55 of 69 patients). There was no worsened tinnitus among patients with preoperative tinnitus. However in one patient without preoperative tinnitus, moderate (60 dB) sensorineural hearing loss occurred. He reported tinnitus complaint postoperatively. Preoperative tinnitus frequencies were as follows: 20 patients with low pitch tinnitus, four patients with middle pitch and 32 patients with high pitch tinnitus.

Thirty-four of 56 (60.7%) patients had a favorable outcome with Group I (28 patients–50%) or Group II (6 patients–10.7%) tinnitus status. Twenty-two of 56 (39.3%) patients had unchanged (Group III) tinnitus status.

**Figure 1:**
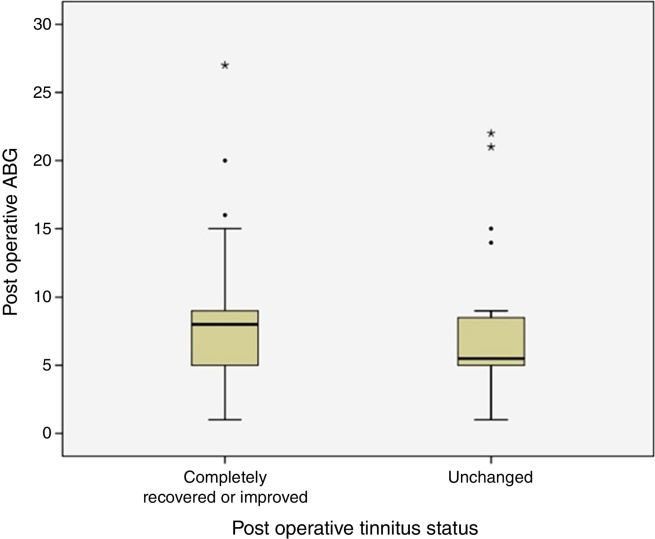
Comparison of postoperative tinnitus status and postoperative average pure tone threshold ABG levels has been demonstrated (ABG, Air-Bone Gap).

Postoperative ABG was smaller than 10 dB in 44 (78.6%) of 56 patients with preoperative tinnitus. ABG was between 10 and 20 dB in 9 (16%) and greater than 21 dB in 3 (5.4%) patients. None of these patients had postoperative sensorineural hearing loss. Preoperative and postoperative frequency specific ABG values were given in Table 1. Relationship between postoperative tinnitus status and postoperative average pure tone threshold ABG levels were shown in Fig. 1. There was no statistically significant difference between postoperative average pure tone thresholds ABG levels and tinnitus status (*p* = 0.213).

**Table 1:** Mean frequency specific preoperative and postoperative Air-Bone Gap (ABG) values.

Frequency (kHz)	Mean ABG	SD	*p*-Values
0.5 Preop	46.73	10.79	<0.001
0.5 Postop	8.84	7.31	
1 Preop	41.92	10.48	<0.001
1 Postop	9.03	7.98	
2 Preop	35.96	9.7	<0.001
2 Postop	7.4	6.52	
3 Preop	35.57	9.73	<0.001
3 Postop	8.17	6.26	

SD, standard deviation; Preop, preoperative values; Postop, postoperative first year values.

Preoperative tinnitus frequency and postoperative tinnitus status was demonstrated in Table 2. There was a statistically significant relationship between preoperative tinnitus frequency and postoperative tinnitus improvement (*p* = 0.003), low pitch tinnitus had a more favorable outcome compared to high pitch tinnitus (*p* = 0.002). There was no statistically significant difference between low and middle (*p* = 0.22), or high and middle pitch tinnitus (*p* = 0.812) regarding postoperative tinnitus improvement.

**Table 2:** Relationship between tinnitus frequency and postoperative tinnitus status.

	Tinnitus		
Subjective tinnitus frequency	Improved or completely recovered	Unchanged	*p*-Value
High frequency	14 (43.8%)	18 (56.2%)	0.003
Middle frequency	2 (50%)	2 (50%)	
Low frequency	18 (90%)	2 (10%)	

**Figure 2:**
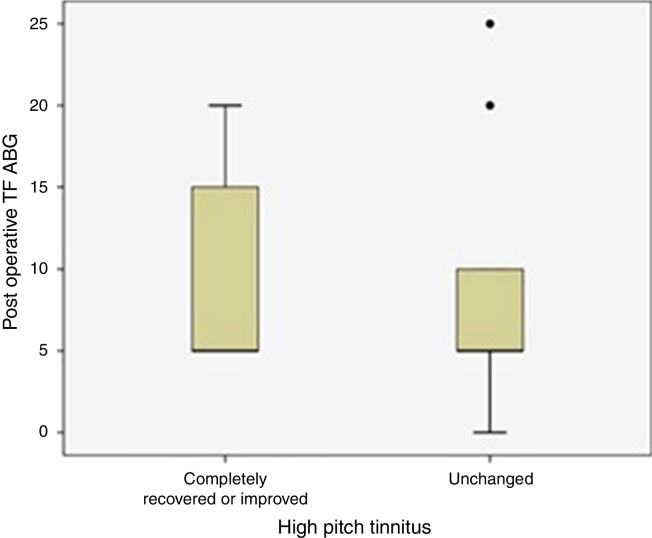
Comparison of high pitch tinnitus status and postoperative ABG values at tinnitus frequencies was shown (TF ABG, Tinnitus Frequency Air-Bone Gap values).

Thirty-two patients with preoperative high frequency tinnitus were evaluated for ABG closure rate at tinnitus frequencies postoperatively. Postoperative tinnitus status of these patients was given in Fig. 2. There was no statistically significant difference between postoperative ABG closure at tinnitus frequencies and improvement of high frequency tinnitus complaints (*p* = 0.427).

**Figure 3:**
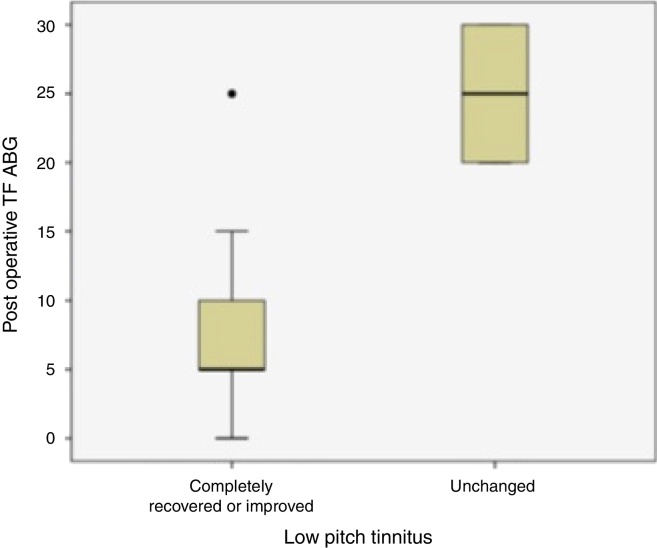
Comparison of low pitch tinnitus status and postoperative ABG values at tinnitus frequencies was presented (TF ABG, Tinnitus Frequency Air-Bone Gap values).

Twenty patients with preoperative low frequency tinnitus were assessed regarding their closure of Air-Bone Gap at tinnitus frequency and postoperative tinnitus complaints (Fig. 3). There was a statistically significant difference between ABG improvement at tinnitus frequencies and tinnitus recovery (*p* = 0.026).

Two sizes of prosthesis were used: 0.4 mm shaft diameter for four of the patients and 0.6 mm for 52 of them. Comparison of Teflon piston diameter and postoperative tinnitus status was summarized in Table 3. There was no statistically significant relationship between prosthesis diameter and postoperative tinnitus status (*p* = 0.641).

**Table 3:** Relationship between Teflon piston diameter and tinnitus status.

	Tinnitus		
Teflon piston diameter (mm)	Improved or completely recovered	Unchanged	*p*-Value
0.6 mm (*n* = 52)	32 (61.5%)	20 (38.5%)	0.641
0.4 mm (*n* = 4)	2 (50%)	2 (50%)	

## Discussion

In the current study, preoperative tinnitus was present in 81.1% of patients with OS; the overall success rate of our stapedotomy surgery was 78.6% for these patients. The tinnitus incidence was reported as 56–84.5%^2^, ^4^ and the success rate of the surgery was shown as 56–92% in previous studies.^4^, ^6^, ^8^, ^15^

Thirty-four (60.7%) patients with tinnitus completely recovered or improved; however, tinnitus was unchanged in 22 (39.3%) patients in our study. The tinnitus had not worsened in any patients postoperatively. The favorable (completely recovered or improved) tinnitus rate has been reported as 40–96% postoperatively in previous manuscripts.^9^, ^15^ Lima Ada et al.^15^ and Szymański et al.^4^ also reported that no patients had worsened tinnitus postoperatively.

In our study, improvement of tinnitus was more pronounced in preoperative low pitch tinnitus compared to the high pitch ones after stapes surgery (*p* = 0.002). The exact pathophysiology of tinnitus is unknown in patients with OS. The efferent auditory pathway has a modulatory effect on the outer hair cells of the Corti organ, which can buffer or amplify the message coming from the brain.^5^ When the auditory stimulus decreases, the central nervous system compensates by increasing the sensitivity of the outer hair cells^16^ and generates a phantom auditory perception as tinnitus. Heller et al.^17^ and Del Bo L. et al.^18^ showed that healthy adults with normal hearing reported tinnitus generation after staying in an anechoic chamber for a long time. The decrease in the inner ear fluid vibration in patients with conductive hearing loss, such as that in OS, diminishes the afferent stimulus of the central auditory pathway. The decreased afferent stimulus in turn decreases the suppressive effect of the efferent pathways on the Corti's organ. Efferent pathway dysfunction is one of the possible mechanisms of tinnitus perception in patients with conductive hearing loss.^5^, ^16^ Causse et al.^5^ stated that the signal amplification mechanism of outer hair cells causes a low pitch tinnitus rather than a buzzing or engine-like noise in cases with conductive hearing loss. When the vibrations of the inner ear fluids are re-established surgically and the hearing loss is improved, the low pitch tinnitus mostly recovers. Our findings and those of several previous studies^6^, ^19^, ^20^ also support this idea. However, Gersdorff et al.^7^ and Ayache et al.^8^ could not find any correlation between the postoperative tinnitus status and the preoperative tinnitus frequency.

Although we had a successful hearing outcome, postoperative tinnitus recovery was not related to the postoperative ABG closure (*p* = 0.213). This finding was supported by Ramsay et al.,^6^ Lima Ada et al.,^15^ Szymański et al.^4^ and Gersdorff et al.^7^ They found no correlation between surgical success and postoperative tinnitus status, either. Recently, Bast et al.^9^ claimed that the success of surgery had an impact on the preoperative compensated tinnitus, whereas the non-compensated tinnitus did not resolve despite a successful surgery. The results of Glasgold and Altmann^21^ and Sparano et al.^22^ did not support our findings, and a poorer postoperative hearing status was more likely to have an unfavorable tinnitus outcome in their studies.

Tinnitus accompanies 70–85% of cases with hearing loss caused by different pathologies of the auditory system.^23^ The pitch of tinnitus often correlates the hearing loss frequencies.^24^ A loss or a decrease in cochlear inputs causes a re-organization and over-signaling of the hearing loss frequencies in the central pathways. For this reason, the pitch of the tinnitus is perceived close to or within the hearing loss frequencies.^25^ From this point of view, we assessed the effect of ABG closure at tinnitus frequencies on the tinnitus outcome in OS patients. We found that when the tinnitus pitch slid to lower frequencies, ABG closure at tinnitus frequency increased the chance of tinnitus improvement. This finding shows that the pathogenesis of high frequency tinnitus in OS patients is a different factor rather than the fixed stapes footplate.

The surgical technique used during OS surgery is mostly accepted to have an impact on the tinnitus outcome. Sakai et al.^19^ showed that a more traumatic surgery such as total stapedectomy had an unfavorable tinnitus outcome compared to partial stapedectomy or stapedotomy. In the study of Gersdorff et al.,^7^ the prognosis of tinnitus was better after stapedotomy compared to partial stapedectomy, and the use of argon laser during surgery did not change the outcome. Ayache et al.^8^ opposed this finding; they found no difference between the surgical techniques and the postoperative tinnitus outcome. Recently, Bagger-Sjöbäck et al.^3^ reported that OS surgery caused high frequency hearing loss beyond 8 kHz and that the tinnitus outcome was directly related to this surgical trauma-induced high frequency hearing loss. However, the findings of Ramsay et al.^6^ did not support this report, and for patients with high frequency hearing loss in the postoperative period due to surgical trauma, the tinnitus outcome was not different compared to the rest of the patients. According to our results, a smaller perforation in the oval window and the use of a smaller diameter Teflon piston did not change the tinnitus outcome, but this finding may be spurious due to the low number of patients with prosthesis of 0.4 mm diameter in our series.

## Conclusion

Stapes surgery not only re-establishes the hearing of patients with OS, but also resolves the tinnitus complaint in most of the patients. The complaint of low frequency tinnitus is more likely to be resolved after surgery. High pitch tinnitus may not resolve even after closure of the ABG at tinnitus frequencies. All otosclerosis patients with the complaint of tinnitus must be evaluated for their tinnitus frequencies preoperatively. The patients with high frequency tinnitus must be informed that their tinnitus complaint may not resolve despite a successful improvement in hearing.

## Conflicts of interest

The authors declare no conflicts of interest.
